# What Should Be the Character and Policy of Dental Journalism?

**Published:** 1898-07

**Authors:** Louis Jack

**Affiliations:** Philadelphia


### WHAT SHOULD BE THE CHARACTER AND POLICY OF DENTAL JOURNALISM?[1]

BY LOUIS JACK, D.D.S., PHILADELPHIA.

It may be stated that the periodical literature of any body or association of men has a great influence upon its advancement and the individual development of those who sustain this interest. This kind of literature furnishes the stimulus to development, since it comes more closely in touch with the individual and tends also to unite the units of an association into a concrete body. Community of interest is exerted, material benefits are promoted, and whenever the policy is liberal and the tone of the journalism is high the general standard becomes elevated. This is more particularly the case with the liberal professions, because with these a broader spirit is expected and a higher estimation of ethical principles is necessary to be sustained.

Books written upon subjects connected with professional matters, or special scientific fields of endeavor, enlighten the student, but have no tendency to unite those interested into the associated effort. They do not weld men to each other, since the appeal is to the intellect only.

The periodicals of a profession, on the contrary, have the tendency to open up living subjects, and are deficient of animating power only when they fail to stimulate moral force and the inculcation of broad ethical standards.

---

[1] Read before The New York Institute of Stomatology.

We must in the final analysis judge each aggregation of men by their special literature, and more markedly by those elements which appeal to questions of right.

Dentistry, from the time of its first appearance in the days of Hippocrates and Galen to the early part of the present century, was pursued as an empirical art, growing slowly by the faint leakage from one isolated mind into another similarly situated. The darkness of ignorance was then general, as there was no adequate means for the dissemination of knowledge. In this country a spirit of improvement of the condition of dentistry began to move upon the existing chaos in the third decade. It appeared among those who were graduates or students of medicine, of whom there were a goodly number who had directed their attention to the field presented by the diseases of the teeth. Those practising in America at that time were composed of two classes,—those just mentioned, who were generally highly intelligent and of great earnestness; and an indefinite proportion of charlatans and itinerants, who went from place to place and house to house soliciting patronage.

The importance of the growing pursuit and its future interests led the qualified class to become associated first for the publication of a periodical devoted to the dissemination of dental science and art.

An appeal was made for the publication of a dental journal, which resulted in the establishment of the *American Journal of Dental Science*, the first issue appearing in 1839. The number of persons becoming responsible for the movement was one hundred and seventy-four, who agreed to take four hundred and eighty-seven copies at five dollars each.

Thus was established the first dental journal to appear in the world. It was strictly professional in character, and maintained in freedom of any outside impediment for many years.

The establishment of the *American Journal of Dental Science* was naturally followed by the organization in 1840 of the American Society of Dental Surgeons. This association was composed principally of those interested in the journal,—editors, contributors, and subscribers.

It will be noticed, in an examination of the events of the time, how intimate the relations of these two movements were.

These events had a marked effect. They were commendably noticed by the London *Lancet* and elsewhere.

It may be irrefutably stated that this was the initiation of a step forward, the effects of which were wide-spread, and which culminated in the present system of dental education and in the establishment of dentistry as a profession upon a secure basis.

The single-mindedness, intellectual force, and moral steadiness of the leaders of these movements produced a great impression upon the mass engaged in practice. The evils of the dental practice of that period were met by vigorous denunciation. The particular evils of those times were the general ignorance of the mass who were practising and the disposition of many to withhold their knowledge of modes of practice from their fellows. The leaders were, from the influence of the medical profession and medical ethics, naturally liberal, and indisposed to accept personal advantage of any advanced methods of practice or of their inventive efforts. The present evils which have had a demoralizing influence upon the dental profession did not then exist.

In 1843 and in 1847 the two dental trade-houses then in existence, conceiving the advantage it would be to them, took the liberty of publishing each a dental periodical, by which they could secure a hold upon the rising profession, thus combining the dissemination of methods of practice and the promotion of their own interests by conveying description of their goods along with the literature contributed to them. The evident result, if not the intention, was the ultimate supplanting of the *American Journal* by offering the new journals at a much lower price. The *American Journal*, not having been pecuniarily profitable, was at length displaced as an independent journal.

Since that time journals independent of trade influences and trade support have been few and their course has been precarious. For obvious reasons, however strong the sentiment which called them into existence, they have had serious impediments to contend with, among which may be stated insufficiency of capital and indifference on the part of the profession from the absence of an active *esprit-du-corps*.

As the dental trade-houses have increased in number and activity, so have the journals connected with these houses been multiplied, for the reason that the cost of advertising in the journals of a rival house being nearly prohibitive, the necessity with each has been to go into the publishing business to promote trade.

It must be admitted that some of these journals have been of excellent character, and have been of immense service to the dental profession. Others have been very indifferent, and have done much harm from their low tone and the incapacity of their projectors.

Along with this experience in the past three decades have grown up several evils which have not been counteracted by the trade journals. I have reference to *the great prevalence of dental patents, the dispensation and use of secret formulæ, and the organization and injurious consequences of the Dental Trade Association.*

The two first stated evils which are damaging to the growth and prevalence of true professional spirit have been encouraged and assisted by the trade-houses, and have not been counteracted by the articles and editorials of their journals.

Here it may be stated that the sinister influence of trade principles are inimical to the high standards and sentiments which should be the governing impulse of professional men.

The intolerance of the trade-houses has gone even further than the encouragement of these injurious policies, for they have endeavored in some instances to control the organization of dentists for protection against these transactions, as was evident by the direct interference with the efforts of the profession to organize for defence when the Cummings patent was resisted, and, again, the opposition has been open as well as covert to counteract the Dental Protective Association.

It comes with ill grace for dental journals to profess devotion to the interests of the profession when they fail to consider favorably those matters which are fundamental to the animation of professional ethics.

It is probably too late for reform to be expected of the trade-houses in the direction of discouraging the patenting by dentists of such simple matters as form the staple of the generally patented affairs applicable to dentistry, or to cease the dispensation of secret formulæ, or for their journals to discredit them.

In view of the wide-spread toleration of unprofessional conduct in the directions indicated, which are alarming to those whose earnest desires are to uphold the honor and dignity of the dental profession, there appears to the writer to be but one creditable position for every journal which professes to be devoted to the interests of dentistry, which is to counteract these two evils by constant references to the danger that is involved in them. Neither by favor nor by silence is it consistent with our general good that any interest should promote the extension of these baneful influences.

As the subject of patent claims and the impropriety of a professional man taking advantage of the privilege of the patent-office to put his profession under tribute to the establishment which purchases his claim is to occupy the attention of other speakers, I shall not take your time further than to lay down the broad statement that it cannot be considered a professional act for one to make such a use of the small ideas involved in the mass of dental patents.

The instruments of surgery present a much larger evidence of inventive talent than is shown in the appliances of dentistry, and you are all aware that any attempt upon the part of a general surgeon to secure such personal advantage would howl him out of any medical society in the land.

The dental profession should impose the same requirements, and no journal should fail to defend the proposition that the sheet-anchor of professional respectability is the same liberality in respect to patent restrictions as is the basis of medical ethics.

The dental supply-houses have been largely responsible for the prevalence of the patent evil because it has been grist to their mills. While they have been fattening on the consequent tribute, their influence and temptings have had a corresponding deadening influence upon professional spirit. Their journals have encouraged by silence or by direct argument the propriety and justice of this practice. From Hill's stopping, which started the greed and set the pace in this direction, to the patenting of instruments, and at last to patenting processes, has been a steady decline.

The evil of secret formulæ has been a greater injury to our standing, and the use of such things a greater mark of indolence and unprofessionality, than patenting. It is more demoralizing, since it is a direct subscription to infamy each time one uses or purchases a secret preparation or proprietary medicament.

There can be only one course for the professional journal to pursue that has any claim to the patronage of our profession, which is to decry the practices which have grown up to such great proportions in the last two decades. This practice should be attacked at all points, and the dental societies should pledge, if need be, their members to avoid this mischievous course and to advocate the use of rational prescriptions.

One of the uses of the schools and societies is to instruct the student to carefully prescribe according to the indications, and the policy of dental journals should be to sustain the schools and associations in this direction.

There is another subject which affects the material interests of those practising dentistry which has not received any attention on the part of the dental journals.

The trade journals avoid any allusion to it because they are silent promoters, and the independent journals have refrained for the reason probably that to discuss it would give offence. I allude to the Dental Trade Association, which is an organization of some of the leading supply-houses for the purpose of restricting competition and maintaining the price of their products. The members of this association are pledged under penalty to buy and sell their manufactured articles only of and to one another, any dealer outside of the association wishing to purchase of its members being required to pay the retail price.

It is not difficult to perceive that these principles of this association are restrictive of trade, and, being destructive of fair competition, tend to prevent lowering of the selling price of goods, and also by lessening the competition between the houses composing the association restrict as well the improvement in many lines of goods.

You have all had illustration of how in some cases when outside competition has become effective, notwithstanding the efforts of the association to cripple the competitor, the goods have fallen to one-half or one-third their former price. Neither have they hesitated to destroy outside competition by attacking a special industry by cutting down the price to a minimum profit. To show how severely this association bears on the dealer or manufacturer outside of it, it is only necessary to state an illustration: A large manufacturer of gold-foil found he could not sell his gold in large quantities unless he connected himself with the Dental Trade Association. He therefore yielded to the inducement.

He had a customer outside the association to whom he had been making annual sales of nearly ten thousand dollars' worth of gold. When this customer made afterwards an order, the manufacturer was most regretfully obliged to answer that under the rules of the association he was required to refuse the order. This relation I had in the language of the retail dealer, with the copies of the correspondence.

Here is an abuse of trade, where it were well wished we had some of the courage of the recently deceased editor and manager of the London *Lancet*, who for many years carried on a combat against those who abused the calling of medicine, and who, attacked on all sides, came out triumphant at last, clearing the atmosphere about the practice of medicine from the degrading impositions and influences which had lowered it.

It requires no argument to show that the Dental Trade Association is an injury to every man practising dentistry. It means that the prices of the manufactured article he uses, from the dental chair to the barbed broach, are much higher than if there existed free competition. It also is an impediment to breaking down the evils of the patent system in dentistry, and supports the procurement and maintenance by their members of questionable patent claims.

There is little hope of the journals connected with the members of this association raising hand or using word to help check the degeneration of dentistry in respect to the evils complained of.

Unless the trade journals will become aroused to resist the degrading tide, professional respect will require us as a body to defend and conserve the interests of the so-called independent journals whenever they appear and wherever they have any footing.

It will then remain for the members of the dental profession who are sensitive to the above-stated abuses to establish and support journals independent of trade influences and which will stand firm for its higher and lasting interests.

The correct development of the dental profession in those matters which give it dignity and self-respectfulness requires the existence and maintenance of some journals which are above trade influences.

Such enterprises are a necessity as a controlling balance over the disturbing influences which hamper us. They also subserve the general good by stimulating better endeavor, and ultimately, if properly supported, must obtain controlling power. It should be kept in view that trade interests come and go as circumstances and fortune lead them, but the dental profession will persist and gather strength, since, like all institutions, its life will be continuous. In the long event its interest must prevail over the oppression and restrictions of personal force. We may be assured that at length the serving body cannot dominate the master power of an organized body of earnest men. This indicates that an appreciation of the necessity for the integrity of our profession, combined with love of it, like the sentiment of patriotism, will overcome all opposition.

